# Invited papers

**DOI:** 10.1054/bjoc.2001.1916

**Published:** 2001


*British Journal of Cancer* (2001) **85**(Suppl 1), 1–11
© 2001 Cancer Research Campaign
doi: 10.1054/ bjoc.2001.1916, available online at http://www.idealibrary.com on

### CT1 CONTINUOUS VS INTERMITTENT CHEMOTHERAPY FOR ADVANCED COLORECTAL CANCER, PRELIMINARY RESULTS OF THE MRC CR06B RANDOMISED TRIAL

T Maughan on behalf of the MRC Colorectal Cancer Group and all the participants Cancer Division, MRC Clinical Trials Unit, 222 Euston Road, London, UK

A survey of UK clinicians suggested that there was no consistent policy regarding the duration of treatment for patients receiving chemotherapy for advanced colorectal cancer. Patients who were responding or had stable disease after receiving 12 weeks of de Gramont, Lokich or Raltitrexed therapy were therefore randomised to either 'continue' therapy until progression, or 'stop', re-starting on the same therapy on progression.

The trial was closed at the end of August 2000, when 354 patients had been entered from 42 UK centres in 4 years.

The median age of patients was 64 years, 64% were male, 41% WHO PS grade 0, and 45% grade 1, 65% had colon cancer, and 40% had responding disease, and these characteristics were well-balanced between the two policies. Of the 178 patients allocated to 'stop', 39% re-started treatment after a median of 134 days, mainly due to disease progression. Median time on re-started treatment was 83 days. The 'continue' group remained on treatment for a median of a further 91 days, stopping for progression (44%), toxicity (15%), or clinician or patient decision (35%). Similar proportions of patients on both groups received second-line therapy. Patients on 'continue' experienced significantly more serious adverse events and toxicity, and using patient-assessed EORTC QLQ-C30 and HADS reported significantly worse quality of life (QL).

There was no clear evidence of a difference in progression-free survival (HR 1.16 95% CIs 0.92–1.45, $P = 0.21$) or overall survival (HR 0.87 95% CIs 0.68–1.12, $P = 0.28$). From randomisation (after an initial 12 weeks of chemotherapy), median, and estimated 1 and 2-year survival were 11.8 and 11.2 months, 47% and 44%, and 18% and 14% for 'stop' and 'continue' respectively.

The result of this trial, that there is no clear evidence of a benefit in continuing therapy indefinitely, and that there appears to be a gain in QL for the 'stop' policy, provides an evidence base for stopping chemotherapy after 12 weeks.

### CT2 IV VS IHA 5FU/LEUCOVORIN FOR COLORECTAL LIVER METASTASES: PRELIMINARY RESULTS OF THE MRC CR05/EORTC 40972 RANDOMISED TRIAL

C McArdle on behalf of the MRC and EORTC Colorectal Cancer Groups and all the participants Cancer Division, MRC Clinical Trials Unit, 222 Euston Road, London, UK

Intrahepatic arterial (IHA) therapy should deliver higher doses of drug to the liver with reduced overall systemic exposure. An IHA regimen was designed which used the same drugs and schedule and achieved similar toxicity and steady-state venous 5FU levels as the standard de Gramont IV regimen. Patients with advanced colorectal cancer with metastases confined to the liver were randomised to either IV or IHA. On days 1 and 2 of a 2-week cycle, IV patients received LV 200 mg/m$^2$ IV over 2 h, 5FU 400 mg/m$^2$ 5-min bolus and 600 mg/m$^2$ IV 22-h infusion, and IHA patients received LV 200 mg/m$^2$ IV over 2 h, 400 mg/m$^2$ 15-minute IHA infusion and 1.6 g/m$^2$ 22-h IHA infusion.

The trial was closed at the end of August 2000, when 290 patients had been entered from 16 centres in 6 years.

The median age of patients was 61 years, 70% were male, 65%, WHO PS grade 0, 68% had colon cancer, and these characteristics were well balanced between the two policies. Of the 145 patients allocated to IV, 15% did not receive any allocated therapy, but 77% received 6+ cycles of chemotherapy. Of the 145 IHA patients, 37% did not start, and only 35% received 6+ cycles.

The additional problems in the IHA group were inability to insert the catheter, or infected, leaking, or blocked catheters. Of the patients who did not receive 6+ cycles of IHA therapy, 49% switched to IV therapy. There was no evidence that the centre or year of entry affected the proportion of patients who did not start IHA therapy.

Clinicians reported similar levels of toxicity, and patients, completing the EORTC QLQ-C30 and HADS, reported similar quality of life in both arms.

There was no clear evidence of a difference in progression-free survival (HR 1.21 95% CIs 0.93–1.58, $N = 0.15$) or overall survival (HR 0.97 95% CIs 0.73–1.29, $N = 0.82$). From randomisation median, and estimated 1 and 2-year survival were 13.4 and 14.7 months, 57% and 56%, and 23% and 20% for IV and IHA respectively.

This trial suggests that there is no obvious role for this IHA regimen in the management of hepatic metastatic colorectal cancer.

### CT3 PRELIMINARY RESULTS OF A MRC RANDOMISED CONTROLLED TRIAL OF POST-OPERATIVE IRRIGATION OF SUPERFICIAL BLADDER CANCER

P Whelan, G Griffiths, M Stower, D Wallace, J Hetherington, T Hargreave, P English, P Weston & M Parmar on behalf of the MRC Bladder Cancer Group. MRC Clinical Trials Unit, 222 Euston Road, London, UK

A multi-centre, randomised controlled trial comparing post-operative irrigation with no irrigation was conducted in the UK (MRC BS04 trial). Eligible patients had newly diagnosed Ta or T1 transitional cell carcinoma of the bladder, a WHO performance status of 0–2, no history of other malignant diseases and an expected survival of at least 3 years. All patients received routine transurethral resection to include muscle and those randomised to irrigation received glycine or saline for a minimum of 18 hours. Patients were followed up by routine check cystoscopy at 3 monthly intervals to assess for recurrence and any side effects. From July 1987 to December 1995 a total of 866 eligible patients were entered by 18 centres, 427 were randomised to post-operative irrigation and 439 to no irrigation. A total of 446 patients had a recurrence. A Cox proportional hazards model for time to recurrence by treatment gave a hazard ratio of 0.83 (95% CI 0.69–1.00 $P$ value = 0.05) in favour of post-operative irrigation. This translates to an absolute improvement in 2-year recurrence-free rate of 6%, 51% with irrigation and 45% with no irrigation. There was no evidence that the effect of irrigation on time to recurrence was larger or smaller in any of the patient subgroups. A total of 302 patients died. A Cox proportional hazards model for survival by treatment gave a hazard ratio of 0.91 (95% CI 0.72–1.14 $P$-value = 0.40). There was only 1 case of increased frequency reported in each arm and 1 reported case of bacterial infection in the irrigation arm. We conclude that post-operative irrigation is easy to give, has little or no toxicity and has evidence of a benefit in terms of recurrence.

### CT4 PRELIMINARY EVIDENCE THAT AN ORAL BISPHOSPHONATE CAN DELAY SYMPTOMATIC PROGRESSION OF BONE METASTASES FROM PROSTATE CANCER: FIRST RESULTS OF THE MRC PR05 TRIAL

DP Dearnaley[1], MP Sydes[2] on behalf of the MRC PR05 collaborators, [1]The Institute of Cancer Research and Royal Marsden NHS Trust, Downs Rd, Sutton, Surrey, SM2 5PT, [2]MRC Clinical Trials Unit, 222 Euston Rd, London, NW1 2DA, UK

**Background** Prostate cancer (PCa) most commonly metastasises to the skeleton. Bisphosphonates have been shown to slow development of metastases from myeloma and breast cancer and to modify pain from bone metastases (BM) from PCa.

**Design** Phase III double-blind placebo-controlled randomised controlled trial of oral bisphosphonate in men with BM from PCa, commencing or responding to standard hormonal treatment. The primary endpoint was time to development of symptomatic bone progression or PCa death.

**Treatment** Either (A) 4 tablets/day (2080 mg) of oral sodium clodronate (Loron 520) or (C) 4 tablets/day of matching placebo. Patients (pts) were encouraged to stay on trial medication for 3 years or until the primary trial endpoint had been reached.

**Results** Patients: 311 pts were randomised over 4 years (6/94–7/98): 156A, 155C. Baseline characteristics were well balanced. Median follow-up fopr this analysis is 3 years. Medication & Toxicity: Median time on trial medication was 18 months(m) for A (95%CI 15–21) and 16m (95%CI 12–20) for C.259 patients have stopped trial medication, 29 (13A, 16C) after 3 years of treatment, 155 (65A, 90C) after symptomatic bone progression and 75 (48A, 27C) because of Adverse Events (AEs) or pt preference. AEs were reported more often for A (118AEs/75pts) over C (69AEs/48pts); Relative Risk = 1.79, $P = 0.0014$; and more often required modification of trial medication dosage (52 vs 20 pts, $P = 0.0001$). Gastro-intestinal problems and raised LDH were the most common AEs. Primary Endpoint: 202 patients have reached trial's primary endpoint, 93 A and 108 C; Hazard Ratio (HR) = 0.75 (95%CI 0.57–0.99) in favour of A ($P = 0.044$). At 2 yrs, 51% (95%CI 44–59) A and 41% (95%CI 33–49) C had not reached primary endpoint. Median time to primary endpoint is 26m (95%CI 21–31) for A and 20m (95%CI 16–23) for C. Survival: 82 A and 94 C patients have died; HR = 0.80 (95%CI 0.59–1.07) in favour of A ($P = 0.13$). At 2 yrs survival is 66% for A and 59% for C. Median survival is 34 m for A and 27 m for C.

**Comments** These preliminary results suggest that oral clodronate delays symptomatic progression of bone metastases from prostate cancer, when initiated whilst patients are still responding to androgen suppression. Updated results will be presented at the meeting.

### CT5 UKCCCR TRIAL OF TAMOXIFEN AND/OR RADIOTHERAPY FOR DCIS

J Cuzick[1], WD George[2], J Houghton[3] (for the DCIS Working Party), [1]Imperial Cancer Research Fund, London, [2]Western Infirmary, Glasgow, Scotland, [3]University College London Medical School, London, UK

A total of 1701 patients were randomised to tamoxifen (or not) and/or radiotherapy (or not) after complete local excision in a 2 × 2 design. Some patients or centres elected to have only one of the randomisations. Most patients (80%) were aged 50 – 64, and DCIS was detected at mammography. A total of 256 breast disease events have now been observed after a median follow-up of 51 months. 107 of these events are invasive cancer and 143 are recurrent DCIS (6 unknown). Radiotherapy led to a 61% reduction in ipsilateral events (95% CI, 38–75%) and had no effect on contralateral events. Tamoxifen produced a non-significant 18% reduction ($P$ = 0.13) in all events. Recurrent DCIS was reduced by 33% ($P$ = 0.02), but there was no effect on invasive cancer.

Other cancers and deaths from other causes were similar in all arms.

### CT6 UKCCCR TRIAL OF EPIRUBICIN AND CYLOPHOSPHAMIDE (EC) VS EPIRUBICIN AND TAXOL® (ET) IN THE FIRST LINE TREATMENT OF WOMEN WITH METASTATIC BREAST CANCER (MBC)

J Carmichael on behalf of AB01 collaborators, MRC Clinical Trials Unit, Cancer Division, 222 Euston Road, London, NW1 2DA, UK

In MBC, Taxol® is active as a single agent in anthracycline failures or when combined with anthracyclines in previously untreated patients. The AB01 trial was a prospectively randomized study comparing the efficacy of six cycles of EC (75 mg/m$^2$ and 600 mg/m$^2$) vs ET (75 mg/m$^2$ and 200 mg/m$^2$) every 3/52 for the first line treatment of MBC. Between 1996 and 1999, 705 patients were randomized at 62 UK centres. Pretreatment characteristics were well balanced between groups, with a median age of 54 years. WHO, PS 0–1 in 84%; visceral metastases were present in 90% with measurable disease in 88% of patients. Median months from initial diagnosis to randomization was 35.3 monthly. Prior hormonal treatment was administered to 72% and adjuvant chemotherapy to 54% (14% anthracyclines). 70% of patients in both arms received all six planned cycles, with epirubicin dose intensity equivalent in both arms. Excluding alopecia, 37% of EC and 46% of ET treated patients had grade 3/4 toxicities during treatment. Severe mucositis (6% vs. 2% $P$ = 0.02) and neurotoxicity (5% vs. 1% $P$ = 0.003) were observed more frequently in patients receiving ET. Severe infection occured in 11% of EC and 14% of ET treated patients. A greater proportion of ET treated patients achieved objective remissions as best response to treatment (67% vs. 56%). With a median followup of 15 months; 88.5% of patients have progressed or died. Median progression-free survival is 6.7 months for EC and 6.5 months for ET (comparing Kaplan–Meier curves, logrank $P$ = 0.72). Median overall survival is 13.8 months for EC and 13.7 months for ET (Logrank $P$ = 0.92). QoL assessed by FACT-B was similar for both arms during treatment. In summary, EC and ET had similar progression-free survival and overall survival when used first line in MBC.

### CT7 THE LONG-TERM IMPACT OF A WATCH AND WAIT POLICY VS IMMEDIATE SYSTEMIC TREATMENT FOR ASYMPTOMATIC ADVANCED STAGE INDOLENT NON HODGKINS LYMPHOMA: RESULTS OF A BRITISH NATIONAL LYMPHOMA RANDOMISED TRIAL

KM Ardeshna, P Smith, DC Linch on behalf of the BNLI, The CRC and UCL Cancer Trials Center, London WIN, UK

The inability of either single alkylating agent chemotherapy or aggressive combination chemotherapy to cure low grade lymphomas, even if combined with radiotherapy, has questioned the necessity of immediate treatment in advanced stage, histologically non-aggressive (low-grade [LG]) non-Hodgkins lymphoma (NHL). Initial studies comparing the outcome of patients with asymptomatic advanced LGNHL who were either treated immediately or carefully followed without initial treatment have been limited by small patient numbers and short periods of follow up. This latter factor is of particular importance in view of the indolent nature of these diseases and the resulting long median survival times. Here we describe the outcome of 309 patients with non-aggressive, advanced (stage III and IV), low-grade NHL who were recruited between 1981 and 1990 into a British National Lymphoma Investigation trial. 158 patients were randomised to receive immediate systemic therapy with oral chlorambucil (Clb), the remaining 151 patients were randomised to an initial policy of observation (with or without radiotherapy to symptomatic areas where necessary), with systemic therapy being delayed until disease progression (Obs). The median length of follow up to date is 14.4 years. There was no significant difference in overall survival or cause specific survival between the two arms [Median OS: Clb = 5.9 years, Obs = 6.7 years ($P$ = 0.68); Median CSS: Clb = 9 years, Obs = 8.8 years ($P$ = 0.43)]. In a multivariate analysis age < 60 years and stage III disease confered a significant survival advantage (OS and CSS). Surprisingly, 14% of patients in the observation arm remain alive and have still not received chemotherapy, having been followed up for a minimum of 10 years. Once treatment was initiated in the Obs arm 30% achieved a clinical CR, which was less than the CR rate of 63% in the Clb arm. In conclusion, an initial period of observation when compared with immediate systemic treatment does not confer a survival disadvantage, and indeed this approach may result in significant numbers of patients not requiring any systemic treatment for over 10 years.

### S1 THE CANCER GENOME PROJECT: SYSTEMATIC SEARCHES FOR MUTATIONS IN HUMAN CANCER

M Stratton, Institute of Cancer Research, Sutton, Surrey SM2 5NG, UK

The advent of the human genome sequence will facilitate the identification of genes that are somatically mutated and implicated in the genesis of human cancer. In part this will be through empowerment of conventional approaches to gene localisation and positional cloning. However, these strategies have their limitations, and ultimately genome wide searches for mutations may be required. The aim of the cancer Genome Project is to develop high throughput mutation detection platforms to acheive these aims. Projects that are underway are designed to detect homozygous deletions, small intragenic mutations (base subsitutions and small insertions and deletions) and chromosomal rearrangements in cancer cells. These are all currently based upon adaptation of conventional mutation detection approaches to very high throughput application. Results from the first studies searching for homozygous deletions will be presented. Homozygous deletions have been detected using a PCR-STS approach coupled to non gel-based detection of products using a fluorescence based assay. These studies show that many homozygous deletions exist at a substantial number of loci in the human genome. Identifying which of these deletions are targetting tumour suppressor genes, which are in zones of fragility (and the underlying reasons for the fragility) and which reflect noise or polymorphism is the next challenge. Ultimately the aim of these studies will be to provide a comprehensive evaluation of the numbers, types and patterns of mutation in the full spectrum of human cancer.

### S2 THE INFLUENCE OF CANCER TREATMENT ON QUALITY OF LIFE (QL) AND COGNITIVE FUNCTION

IF Tannock, Princess Margaret Hospital and University of Toronto, Toronto, Canada

Many clinical trials have the goal of improving palliation for patients with cancer, but they do not often measure palliation. Rather they use measures such as tumour shrinkage, with the implicit and potentially false assumption that this is a valid surrogate. Palliation implies improvement in the duration and/or quality of survival, but improvements in the duration of survival remain elusive. Questionnaires are available that allow QL to be assessed in clinical trials, with reasonable psychometric properties. However, QL is rarely a primary endpoint and it is seldom applied with the same rigour as more traditional endpoints. Few trials have adhered to the following principles:

1. A primary endpoint relating to QL should be defined that is relevant to the patient. This might be a global QL score or a measure of the dominant symptom. Changes in other aspects of QL can be regarded as exploratory.
2. There should be a primary hypothesis about the magnitude of change that is meaningful to the patient. This meaningful change needs to be based on evidence.
3. It is essential to recognise that QL is a property of an individual patient that will change at different rates and in different directions among a population of patients.

A common method for evaluating QL in a clinical trial is to measure a mean score for multiple QL endpoints in the randomised groups at baseline and at a fixed time (e.g. 3 months) later. The groups are then compared for each endpoint and for differences at the 3-month time point. Unfortunately, mean QL has little meaning, enough comparisons are done that some will inevitably appear "significant", and mental torture is involved in dealing with drop-outs and lack of compliance. Instead we need to record changes in the major endpoint of QL in the individual patient and determine if he or she has had a palliative response, and for how long it lasts; here we can learn by analogy from evaluation of tumour response. Principles of using a primary QL endpoint as an individual property in a clinical trial will be illustrated by the Canadian trials that evaluate chemotherapy for patients with symptomatic hormone-resistant prostate cancer.

Measures of QL are now being used to define psychosocial aspects of disease and its treatment that may be as important as the physical effects. Patients receiving adjuvant chemotherapy for breast cancer now perceive side effects such as fatigue and menopausal symptoms due to treatment as more disabling than nausea or vomiting. Also, our recent studies and those of Dutch investigators have detected substantial cognitive changes, which can occur during chemotherapy and they are not rapidly reversible. The results of our pilot and ongoing studies of cognitive changes, fatigue and menopausal symptoms in patients receiving adjuvant chemotherapy for breast cancer will be described.

### S3 NEW WAYS TO EXPLOIT TUMOUR HYPOXIA

M Brown, Department of Radiation Oncology, Stanford University, Stanford, California 94305, USA

Hypoxia in human solid tumours provides attractive opportunities for tumour specific anti-cancer therapy. We have developed a drug, tirapazamine, that is selectively activated to a damaging free radical under hypoxic conditions. This drug is currently showing considerable promise in phase I, II and III clinical trials when combined with cisplatin and/or with radiation therapy. To obtain insight into the mechanism of hypoxic cell killing by this agent we have used a genomics approach using a pool of 4627 individual homozygous deletion strains (representing deletions of all non-essential genes) in the budding yeast *S. cerevisiae*. Each open reading frame is replaced with a cassette containing unique tag sequences that allow rapid parallel analysis of strains in a pool using hybridization to a high density oligonucleotide array (Winzeler et al 1999, Science 285:901). We first tested this technique using UV radiation as the test cytotoxic agent and identified essentially all the nonessential genes previously shown to affect sensitivity to this agent. Using this system with tirapazamine we have identified genes that when deleted confer sensitivity to the drug. We find that homologous recombination following DNA damage is the important lesion effecting sensitivity to tirapazamine both in yeast and in mammalian cells. In the second approach to exploit tumour hypoxia we have transformed a nonpathogenic spore forming obligate anaerobe, *C. sporogenes*, with a plasmid encoding the prodrug activating enzyme, cytosine deaminase. Following IV injection of recombinant spores this enzyme is expressed specifically in transplanted mouse tumours. We further show that systemic administration of the non-toxic pro-drug 5-fluorocytosine produces significant anti-tumour activity when combined with systemic administration of the recombinant spores. Both of these approaches show promise for anticancer therapy.

### S4 MEASURING TUMOUR HYPOXIA IN THE CLINIC

C West, Paterson Institute, Christie Hospital NHS Trust, Manchester, M20 4BX, UK

The current resurgence of interest in hypoxia stems from studies... showing the prognostic significance of oxygen electrode measurements of tumour oxygenation for outcome following either radiotherapy or surgery. This has been accompanied by increasing awareness that a large number of angiogenic and metastasis-promoting proteins are induced under prolonged hypoxia. There is, therefore, a renewed drive towards finding tests for measuring tumour hypoxia that can be applied to a wider range of tumours than possible using oxygen electrodes. In recent years we have assessed clinically a variety of methods for their ability to measure tumour hypoxia: oxygen electrodes and an Eppendorf $pO_2$ histograph, the hypoxia specific probe pimonidazole, dynamic MRI, vascularity, and the expression of hypoxia inducible proteins in tumour sections (VEGF, PD-ECGF, CA9, Glut-1). Martin Brown first suggested the existence of two types of hypoxia, acute and chronic, that was subsequently confirmed experimentally. The therapeutic opportunities arising from the existence of hypoxia in tumours are likely to depend on the type of hypoxia present. We hypothesised that different methods for measuring hypoxia will measure different types of hypoxia.

Carcinoma of the cervix treated with radiation alone is an excellent tumour model for trying to unravel the molecular consequences and therapeutic opportunities of acute versus chronic hypoxia. Acute hypoxia caused by the intermitant closure of blood vessels will induce radiation resistance and should be associated, at least for tumours treated with radiation alone, with local failure. In contrast tumours with a high prevalence of chronic hypoxia should have an increased propensity for angiogenesis and metastatic spread. Our clinical studies have shown that different endpoints predict for either local control (oxygen electrode, dynamic MRI, vascularity) or metastasis-free survival (expression of inducible factors) highlighting the importance of understanding the type of hypoxia present in a tumour in order to select the most appropriate treatment.

### S5 GASPING FOR BREATH: DEVELOPMENT OF THERAPEUTIC AND IMAGING AGENTS TARGETING TUMOUR HYPOXIA AND BIOREDUCTIVE ENZYMOLOGY

P Workman, Director, CRC Centre for Cancer Therapeutics, Institute for Cancer Research, Sutton SM2 5NG, UK

The discovery and development of new drugs and diagnostic reagents is increasingly based on the identification of molecular and genomic factors that drive the molecular pathology of human cancer (see Garrett and Workman, 1999 Eur J Cancer 35:2010). At the same time, environmental differences between solid tumours and normal tissues continue to provide alternative opportunities for selective therapeutic intervention. In particular, tumour hypoxia can be exploited by the design of bioreductive agents–prodrugs that are selectively activated under low oxygen conditions or by overexpression of reductase enzymes, such as the *NQO1* gene product DT-diaphorase (see Stratford and Workman, 1998 Anti-Cancer Drug Design 13: 519). I will describe some of our recent work in two areas: 1. Understanding the respective contributions of hypoxia vs. reductase expression in the selective antitumour activity of bioreductive agents (e.g. Sharp et al, 2000 Mol Pharmacol 58:1146); 2. Development of SR4554 as an agent for imaging tumour hypoxia by magnetic resonance (MR) and positron emission tomography (PET) (e.g. Aboagye et al, 1997 Cancer Res 57: 3314). We developed an isogenic model involving comparison of BE human colon carcinoma cells (which contain a disabling polymorphism in the *NQO1* gene) transfected with *NQO1* or control vector. DT-diaphorase enhanced aerobic sensitivity by factors ranging from 7-fold for mitomycin C to 21-fold for indoloquinone EO9 and 118-fold for streptozotocin. With aziridinylbenzoquinone RH1, which is undergoing preclinical development, sensitization by *NQO1* depended on time of exposure. We are using the isogenic BE +/– *NQO1* model to elucidate the precise role of DT-diaphorase in the mode of action of compounds identified from the NCI 60 human tumour cell panel by virtue of a correlation between sensitivity and previously determined DT-diaphorase activity (Fitzsimmons et al, 1996 J Natl Cancer Inst 88; 259). Interestingly, *NQO1* transfection led to a 32-fold sensitization to the ansamycin benzoquinone Hsp90 molecular chaperone inhibitor 17-allylamino, 17-demethoxy geldanamycin (17AAG) (Kelland et al, 1999 J Natl Cancer Inst 91; 1940). Whereas mitomycin C showed no differential antitumour activity towards *NQO1*-translated BE cells grown as a solid tumour xenograft, 17AAG was more active against DT-diaphorase-rich tumours. As a result, we are carrying out *NQO1*-genotyping in our Phase-1 clinical trial of 17AAG. Optimal use of hypoxia-based therapies requires the identification of patients most likely to benefit. SR4554 was designed for this purpose. In addition, it could be useful in monitoring the effects of antivascular and antiangiogenic therapies, and as a general prognostic indicator. We have continued to validate the use of SR4554 by correlating fluorine retention determined by MR with direct oxygen electrode measurement. We used the comparatively well oxygenated P22 rat tumour xenograft and perturbed oxygen levels by various means. A direct correlation was observed. We have now initiated a Phase I clinical trial of SR4554. Initial pharmacokinetic and spectroscopy data are promising and an update will be provided.

### S6 HYPOXIA REGULATED TRANSCRIPTOME – IMPLICATIONS FOR TUMOUR ANGIOGENESIS AND THERAPY

AL Harris, ICRF Medical Oncology Unit, Churchill Hospital, Oxford OX3 7LJ, UK

Hypoxia is common in all types of cancer and associated with poor outcome. It is well recognised to be associated with resistance to radiotherapy and some types of chemotherapy, but independent of any adjuvant treatment it is also associated with poor outcome. This suggests that there is hypoxia regulated programme affecting the biology of cancer in several ways. Recently hypoxia inducible factors 1 and 2 have been shown to be regulated by mutations in the VHL gene and involved in familial and sporadic renal cancer, thus demonstrating the importance of a hypoxia signalling pathway in tumour biology. We have investigated genes regulated by hifl$\alpha$ and hif2$\alpha$ using a variety of approaches including SAGE, GEM and proteomics. This has revealed several new pathways regulated by hypoxia including transmembrane carbonic anhydrases 9 and 12, a novel delta notch signalling pathway in vessels, and upregulation of pro-apoptotic genes such as nip3 and nix. The carbonic anhydrases are upregulated and are associated with poor outcome in several tumour types including breast cancer, cervical cancer, head and neck cancer and lung cancer. The novel delta ligand appears to be activated in tumour endothelium mainly in the arterial side of circulation and since it is extra cellular maybe particularly suitable for vascular targeting. A surprising number of apoptotic pathways were activated by hypoxia and results suggest that escape from pro-apoptotic pathways must be one of the earliest changes in tumour evolution. The potential role of the carbonic anyhdrases in regulating extra cellular pH links long standing observations on the interactions between hypoxia and acidic pH, but suggests that this may be mediated by carbonic acid rather than lactate. Extra cellular locations of the enzymes supplies the target for both antibody targeting and also inhibition of the enzymes to potentiate chemo- or radiotherapy. Over 60 genes that are regulated by hypoxia include many angiogenic factors, cell adhesion molecules, proteases and their receptors as well as glycolysis, iron uptake and ion transporters. Thus, it is understandable that hypoxia should contribute to an aggressive tumour phenotype by induction of such a complex programme and it is difficult to dissociate the role of aggressive tumours inducing hypoxia, by metabolism of oxygen and activating the pathway versus hypoxia switching on a programme in a less aggressive tumour and advancing its behaviour. However, convincing evidence of the importance of the hypoxia signalling pathway has been demonstrated in model systems where blocking the function of hif either by its interaction with CBP300 or with its partner ARNT, have inhibited tumour growth.

Thus, inhibition of hif signalling is a potential new major target to be developed for cancer therapy applicable to all types of cancer and potentially in combination with radiotherapy and chemotherapy.

### S7 THE ROLE OF GENE PROMOTER HYPERMETHYLATION IN THE EVOLUTION OF COLON CANCER

SB Baylin, The Johns Hopkins Comprehensive Cancer Center, Baltimore, MD 21231, USA

During the past several years, it has become increasingly apparent that cancer is a disease driven by epigenetic as well as genetic alterations. The most cell studied aspect of the former is aberrant hypermethylation of gene promoter regions which is a signature for chromatin alterations associated with transcriptional silencing, in cancer cells, of normally expressed genes. This silencing, in turn, can serve as an alternative to coding region mutations for loss of key gene function. These epigenetic abnormalities appear particularly important for the evolution of colon cancer during which aberrant promoter methylation begins early and prior to the development of benign and malignant tumors. A series of genes, including the *estrogen receptor*, actually manifest this change in the colon as a function of aging and these genes are the most frequently hypermethylated in benign colonic polyps and cancers. Another group of genes, including several which are critical to colon cancer evolution such as *p16*, *MLH1*, and *APC*, do not appear to be hypermethylated in normal colon but can evolve this change by the stage of benign polyps. Importantly, epigenetic changes during the progression of colon cancer can be integrally tied to key genetic alterations. Thus, the most frequent cause for microsatellite instability in sporadic colon cancers is loss of *MLH1* expression in association with promoter methylation. A similar loss of the DNA repair gene, $O^6$-*MGMT*, appears to bias tumors towards specific spectra of mutations in the K-RAS and p53 genes. The study of cultured colon cancer cells is providing increasing insight into the basic mechanisms underlying both how promoter methylation silences genes and the determinants of the abnormal methylation patterns methylation patterns themselves. Studies of the TIMP-3, p16, and MLH1 genes has revealed that the transcriptional silencing is mediated by synergy between the methylation and maintenance of the de-acetylated state of histones and possibly other proteins. This chromatin synergy may affect certain groups of genes in concert since a 'hypermethylator phenotype' appears to characterize a subset of colon cancers which includes those with microsatellite instability. The DNA methylation machinery which is responsible for establishing and/or maintaining aberrant promoter methylation is becoming apparent through study of colon cancer cells in which both alleles for the DNA methylation catalysing enzymes, the DNA methyltransferases, have been deleted.

All of these above studies are providing rich potential for translational work to develop the aberrant promoter methylation patterns as sensitive markers for tumor detection and for considering the DNA methylation and histone de-acetylation pathways as targets for new prevention and therapeutic strategies.

### S8 USE OF EXPRESSION ARRAYS TO IDENTIFY NOVEL THERAPEUTICS TARGETS IN COLORECTAL CANCER

K. Millan, Genentech, DNA Way, South San Francisco, CA 94080-4990, USA

**Abstract not received.**

### S9 TOTAL MESORECTAL EXCISION (TME) WITH OR WITHOUT PREOPERATIVE RADIOTHERAPY (RT) IN THE TREATMENT OF PRIMARY RECTAL CARCINOMA

RAEM Tollenaar, E Kapiteijn, CAM Marijnen, ID Nagtegaal, M van den Brink, WH Steup, HJT Rutten, T Wiggers, JHJM van Krieken, JWH Leer, CJH van de Velde, On behalf of the Dutch ColoRectal Cancer Group (DCRCG), Leiden University Medical Center, Department of Surgery, The Netherlands

**Introduction** A major problem in the treatment of rectal cancer is the high rate of local recurrences (20–30% with conventional surgery). The TME-trial was set up: a to document local control of primary rectal cancer when standardised TME-surgery and pathology are applied and; b to investigate whether the local recurrence rate after TME permits the omittance of adjuvant $5 \times 5$ Gy preoperative RT.

**Material and methods** Patients were randomised for TME-surgery alone or preoperative RT of $5 \times 5$ Gy, followed by TME. Surgical quality control took place by means of a monitoring committee of specially trained instructor-surgeons. Pathological examination was also standardised and performed according to the protocol of Quirke.

**Results** From January '96 up to December '99 1530 patients were randomised from 84 Dutch hospitals, and 331 patients from 24 foreign hospitals. Neurotoxicity was the most severe radiotherapeutical side effect reported, but the incidence was low. There were only differences between the randomisation groups with regard to blood loss during operation (larger in the RT-group) and the percentage perineal dehiscence (higher in the RT-group). Local recurrence rate at 2 years was only 5.3% in patients who underwent a local complete resection (median follow-up 25 months).

**Conclusion** The TME-trial has shown that the combination of preoperative, hypofractionated radiotherapy and TME-surgery is safe, and that local control of rectal cancer has improved. The effects of radiotherapy in various subgroups (tumor location, tumor stage and margin status) will be presented.

Furthermore, the role of the pathologist in judging the quality of surgery and the cost-effectiveness analysis of adding pre-operative radiotherapy to standardized TME-surgery will be discussed.

### S10 MOLECULAR GENETICS OF COLORECTALCANCER: IMPLICATIONS FOR MANAGEMENT IN OLD AND YOUNG PATIENTS

Andrew H Wyllie, Department of Pathology, Tennis Court Road, Cambridge, CB2 1QP

A classification of colorectal cancers based upon their patterns of genomic instability is attempted. In patients over 50 years of age, 15% of cancers show microsatellite instability (MIN) – sometimes referred to as the RER+ phenotype. These tumours have been well described by several groups. Usually right-sided and often mucinous, they carry a relatively good prognosis. Most, though not all, are DNA diploid on flow cytometry, and show few chromosome arm imbalances on Comparative Genomic Hybridisation (CGH). Data from multiple samples within single tumours, and from xenografts in SCID mice, prove that the microsatellite instability is continuous, with new alleles appearing throughout the tumours' growth histories. MIN carcinoma cell lines are readily available. In these, 24-colour FISH usually reveals near-diploid karyotypes, but a proportion show unexpected features such as balanced translocations. Of the remaining 85% late-onset tumours, approximately two-thirds are aneuploid, with near-triploid DNA content and many chromosome imbalances on CGH, most commonly 7+, 8p-, 8q+, 13q+, 18q-, 20q+. Other imbalances occur with less consistency but are clearly acquired during tumour growth in situ or as xenografts, demonstrating a true chromosomal instability (CIN). Many available cell lines show this pattern. Their karyotype includes many trisomies and invariably there are unbalanced translocations, at inconstant sites. Approximately one third of all late-onset colorectal cancers, however, fit neither the MIN nor CIN pattern. They are near-diploid, RER- and show few chromosome imbalances on CGH. We refer to these as MACS (for microsatellite and chromosome stable). Clinically, they are moderately differentiated adenocarcinomas and almost all are rectal. So far we have found no examples of this type amongst available cell lines, but do not know if this is because MACS tumours convert to other types in vitro, or because they adapt poorly to in vitro culture. Early-onset cancers (in patients less than 45) show a different distribution, with more than half MIN, and the majority of the remainder MACS. Such young patients, with both MIN and MACS tumours, tend to have positive family histories of cancer – and in patients with MIN tumours there are frequent germline mutations involving mismatch repair genes. Early onset MIN tumours tend to be aggressive and are not consistently right-sided. Exposure of cell lines of different types to ionising radiation and chemotherapy in vitro reveals striking differences in sensitivity, implying that awareness of the type and cause of genomic instability may have profound implications for management.

### S11 REGULATION OF THE RAF-1 PROTEIN KINASE BY PHOSPHORYLATION

A Chiloeches, N Dumaz, M Garnett, L Hayes, Y Light, R Marais, Signal Transduction Team, The Institute of Cancer Research, 237 Fulham Road, London SW3 6JB, UK

The Raf-1 protein kinase couples activation of the Ras proto-oncogene to activation of the ERK family of mitogen activated protein kinases. Ras signalling to the ERKs is important in the regulation of cell growth and has been implicated in the pathogenesis of cancer. The Raf proteins are therefore important regulators of cell growth and a number of compounds that target the activity of these proteins have been developed. Our aim is to gain a full understanding of the mechanisms by which Raf proteins are regulated, as this may lead to a better understanding of the pathogenesis of cancer and may lead to the discovery of important new targets or approaches to the treatment of cancer.

One aspect of Raf-1 regulation that we have been particularly interested in is its regulation by phosphorylation. We have shown that phosphorylation of two residues in the catalytic domain of Raf-1 (serine 338 and tyrosine 341) are essential for its activation. Recently, we have created germ-line mutations of one of these sites (Y341) in the genome of mice, to create mice whose Raf-1 cannot be activated. Surprisingly, these mice develop normally, are fertile and live a normal life span. By contrast, mice that we have created that are null for Raf-1 die during mid-gestation. Thus, it appears that while Raf-1 protein is essential, its kinase activity is not required, suggesting that targeting Raf-1 for the treatment of cancer may be a feasible approach.

We have also been examining the suppression of Raf-1 by agents that lead to elevated cAMP in the cell. We have found that when cAMP levels are elevated, there is a suppression of Raf-1 activity, which is mediated through the phosphorylation of at least three sites on Raf-1. Two of these have previously been mapped by others and are the serine at position 43 and the serine at position 259. In addition, there is a third site, which when phosphorylated also appears to suppress Raf-1 activation by growth factors. We are currently attempting to map this site.

### S12 NEW DRUG DEVELOPMENT – ITS ROLE IN REVERSING DRUG RESISTANCE

SB Kaye, Institute of Cancer Research, Royal Marsden Hospital, Sutton SM2 5PT, UK

The focus in cancer drug discovery is correctly shifting towards rational development of selective agents, often based on aberrant molecular signalling in cancer cells. However, it would be unwise to assume that conventional chemotherapy will not continue to be widely used – indeed its usage is predicted to increase over the next five years. The challenge surely is to optimise the combination of the new with the old. This will require a better understanding of the limitations of current cytotoxic drugs, i.e. the mechanisms underlying clinical drug resistance. Increasingly it is being recognised that this will need careful analysis, e.g. gene profiling using microarray technology, of appropriate clinical samples rather than further work on experimental models.

However, these experimental systems have proved useful in demonstrating that many novel approaches to treatment, including signal transduction inhibitors (either small molecules or monoclonal antibodies) are clearly capable of reversing the resistant phenotype. Examples of successful chemotherapy resistance modulators already in the clinic include Herceptin and C225 (monoclonal antibodies to HER2/ncu and EGFr). A large number of other molecules show similar potential. These include EGFr tyrosine kinase inhibitors, e.g. ZD1839; farnesyl transferase inhibitors, e.g. R115777; molecular chaperone (HSP90) inhibitors, e.g. geldanamycin analogues; proteasome inhibitors, e.g. PS341; cell cycle inhibitors (CDK2 and 4), e.g. flavopiridol; inhibitors of the PI3 Kinase / AKT pathway, e.g. CC1779, and (for the future) inhibitors of histone deacetylation, and of gene hypermethylation (5 aza-2-deoxycytidine).

In each case, the hypothesis is that following DNA damage the balance of cell signal machinery can be shifted away from cell survival and resistance, towards apoptosis and chemosensitivity. Careful clinical development, incorporating proof-of-principle Phase I trials, will test the hypothesis that this can be a tumour selective process, and large scale randomised trials will then be necessary to ascertain the clinical reality of resistance reversal.

### S13 ENHANCING ANTI-MELANOMA IMMUNE RESPONSES

M Harries, Dept. Medicine The Royal Marsden Hospital, Downs Rd, Surrey UK SM2 5PT (formerly at Dept. Immunology, The Windeyer Institute, UCL, London WI)

This paper will describe research conducted by the author in the laboratory of Professor Mary Collins in the Department of Immunology at UCL that led to the award of a PhD in June 2000, together with ongoing work. The work described falls into four sections:

1. In vitro experiements examining the effect of interferon-alpha (IFN-α) on mixed lymphocyte tumour cultures. Using cell-mediated cytotoxicity assays, IFN-α was shown to increase both allogeneic and autologous anti-melanoma CTL generation from peripheral blood lymphocytes stimulated with irradiated primary melanoma cultures.
2. The construction and testing of retroviral vectors able to direct synthesis of bioactive interleukin-12 (IL-12) in target cells. Retroviral vectors were constructed to express IL-12 in which an internal ribosome entry site (IRES) initiates translation of the p40 subunit with the IRES optimally aligned to the initiation codon of p40. Vectors containing an IRES from either polio virus (PV), encephalomyocarditis virus (EMCV), foot and mouth disease virus (FMDV) or murine leukaemia virus (MLV) were compared with a vector expressing human IL 12 as a single protein (Flexi-12; in which the two IL-12 subunits are linked by a peptide).
3. Engineering antigen presenting cells to present optimally processed melanoma antigens for cancer gene-therapy. The Collins lab has developed lenti-viral vectors that are capable of transducing macrophages and dendritic cells (DCs).In an in vitro, model, vectors designed to express modified melanoma antigens targeted for proteosomal degradation were compared to vectors expressing unmodified proteins. Results showed that B cells expressing either the wild-type MAGE-3 protein or the modified constructs did not present an HLA-A2 epitope. In contrast, it was found that HLA-A2$^+$ B cells expressing NY-ESO-1 or Melan-A were lysed by HLA-A2 restricted specific CTL efficiently but that lysis was not enhanced by modifications of the proteins desinged to increase degradation.
4. The identification and characterisation of an internal ribosome entry site (IRES) in Kaposi's sarcoma-associated herpes virus (KSHV) – the first IRES identified in a DNA virus. We have identified and characterised a previously unknown IRES in the bicistronic v-cyclin/vFLIP transcript of KSHV that facilitates translation of the vFLIP message. v-FLIP translation was found to occur from the bicistronic transcript even when v-cyclin translation was disrupted.

### S14 HIGH-THROUGHPUT SCREENING FOR INHIBITORS OF SIGNALING PATHWAYS

RH Shoemaker, Screening Technologies Branch, Developmental Therapeutics Program, National Cancer Institute, Frederick, MD 21702–1201, USA

Resources for high-throughput screening at the National Cancer Institute are being directed towards molecular targets in cell signalling pathways. The success of such a program depends on selection of appropriate targets, establishment of useful high-throughput assays, and conduct of screening campaigns using chemical libraries of adequate chemical diversity. We have focused on use of cell-based luciferase-reporter constructs to probe pathways related to proliferation, differentiation and angiogenesis. Cell-based assays present the advantage of representing targets in a relatively natural context compared to cell-free biochemical or biophysical assays. However, they are more labour-intensive and yield 'hits' which require additional study to fully define the mechanism of drug action. We have utilized the National Cancer Institute's 'Diversity Set' of approximately 2000 compounds for initial screening against several molecular targets. This small library was selected by computer algorithm to provide a representation of the chemical diversity present in the entire chemical repository which has been collected over forty years. The Diversity Set contains many standard anticancer and other drugs, specific biochemical inhibitors, toxins, and compounds with unknown biological activity. For each target, screening of this library at 1 μM has yielded multiple 'hits'. Intersection of hits across screening models can be used to identify compounds with non-specific inhibitory action or may provide suggestions of inhibitory action at common up-stream regulatory elements in the signalling pathways. This growing database is providing a rich environment for chemical and bioinformatic analysis which has the potential to define new structure-activity relationships. Linkage to the publicly available 60 tumour cell line screening database has the potential to define a mechanistic basis for clusters of activity which were previously not understood and can provide insight into additional chemotypes which may be useful for lead optimization.

### S15 CYCLIN-DEPENDENT KINASE INHIBITORS: NEW COMPOUNDS, SELECTIVITY, MECHANISMS OF ACTION AND ANTI-PROLIFERATIVE ACTIVITIES

L Meijer, Station Biologique, BP 74, 29682 Roscoff cedex, France (meijer@sb-roscoff fr)

Cyclin-dependent kinases (CDKs) directly regulate the cell division cycle phases, transcription, apoptosis and neuronal cells and thymocytes functions. Intensive screening has led in the last few years to the identification of several families of chemical inhibitors of CDKs. Some of these compounds display a high selectivity and efficiency ($IC_{50}$ < 5 nM). Many have been co-crystallised with CDK2 and their atomic interactions with the kinase have been analysed in detail: all are located in the ATP-binding pocket of the enzyme. Some novel structures will be presented.

Despite high selectivity, most CDK inhibitors (except purines) are potent inhibitors of glycogen synthase kinase-3 (GSK-3). Whether this GSK-3 inhibitory property is favourable to the anti-mitotic properties of CDK inhibitors will be discussed. An overall method for the determination of selectivity of kinase inhibitors, based on the affinity chromatography purification of targets on immobilised inhibitor, will be presented. This method has led to the identification of unexpected targets.

CDK inhibitors display anti-proliferative properties, they arrest cells in G1 and in G2/M. Furthermore they facilitate or even trigger apoptosis in proliferating cells. In contrast, they protect neuronal cells and thymocytes from apoptosis. This suggest that CDKs may be involved both in triggering and inhibiting apoptosis. The consequences of this dual and conflicting effect need to be evaluated. The combination of CDK inhibitors with other anti-mitotic agents greatly enhances their anti-tumour activity. This will be illustrated by a few examples. The potential of CDK inhibitors is being extensively evaluated for cancer chemotherapy (clinical trials, phase I and II) and will be briefly reviewed.

### S16 ANTI-ANGIOGENESIS TYROSINE KINASE INHIBITORS: FROM THE LAB TO THE CLINIC

L Shawver, Sugen Inc, 230 East Grand Avenue, South San Francisco, CA 94080, USA

**Abstract not received.**

### S17 FARNESYL PROTEIN TRANSFERASE INHIBITORS: PROGRESS ON MOLECULAR MECHANISMS AND IN THE CLINIC

DW End, Dept of Oncology, Janssen Research Foundation, Titusville, New Jersey 08560, USA

Over 10 years have passed since the original description of farnesyl protein transferase (FPT) triggered a race for small molecule inhibitors with the hope of providing a better tolerated targeted therapy of cancer. Although the original hypothesis anticipated that FTIs would provide therapy targeted specifically at the farnesylated Ras oncoproteins and cancers with *ras* gene mutations, the advent of potent small molecule inhibitors revealed that several other prenylated effectors may contribute to the antitumor activity of FTIs. Compelling evidence now exists for a role for RhoB and some farnesylated centromere associated proteins in the antiproliferative effects of FTIs. Upregulation of TGF-β type II receptors with restoration of sensitivity to the growth suppressive activity of TGF-β may also contribute downstream from the prenylated effectors. Consistent with actions on targets other than Ras, the spectrum of preclinical and clinical antitumor activity of FTIs has been shown to extend beyond tumors with *ras* mutations. Also, the antitumor activity of FTIs has been shown to involve significant host tumor interactions resulting in far more than the cytostatic activity, which had been anticipated for this class of compound. These actions include induction of apoptosis and antiangiogenic effects. At least in animal models, robust antitumor effects including tumor regression have been obtained from this enzyme-targeted therapy.

Despite some uncertainty about the downstream cellular effectors for FTIs, the interaction of inhibitors in the active site of the FPT enzyme can be accurately described thanks to the publication of the X-ray structure as well as excellent mechanistic work. The enzyme is a heterodimer with a unique catalytic zinc. Several inhibitors target the zinc and the associated CAAX peptide binding site. Fewer inhibitors have been reported to compete for the farnesyl pyrophosphate binding site of the enzyme.

At the moment, four FTIs have data published from various phases of clinical testing. At least one compound is in Phase III trials, and two to three additional molecules may have entered Phase I. The early published Phase I and II data indicate that the side effects of FTIs are manageable and allow for chronic treatment regimens. Toxicities of myelosuppression and fatigue appear to be specific to the class. Varying degrees of gastrointestinal toxicity have also been reported. Hints of activity have been observed in solid tumors in trials of FTIs as single agents. The activity reported for R115777 in advanced breast cancer (Johnston et al, ASCO 2000) is noteworthy. Significant clinical activity has also been observed in some hematological malignancies such as AML and CML (Lancet et al, ASCO 2000). A number of studies combining FTIs with various cytotoxic regimens are ongoing. The combination of FTIs with radiation appears to be a promising area based upon both preclinical and clinical data. Even after a decade of research, many research opportunities remain in defining the pharmacology of FTIs and their optimal clinical use.

### S18 DISRUPTION OF CALCIUM SIGNALING FOR THE TARGETED INDUCTION OF APOPTOSIS OF PROSTATE CANCER CELLS

SR Denmeade, B Tombal, A Weeraratna, C Jacobsen, SR Khan, SB Christensen, JT Isaacs, The Johns Hopkins Oncology Center, Baltimore MD, USA

Prostatic cancer cells are lethal because they acquire androgen independency for their proliferation and survival. These androgen independent prostatic cancer cells do retain, however, the machinery for apoptosis even though this is no longer activated by androgen ablation. One method for activating this apoptotic cascade is to disrupt the regulation of the intracellular free calcium $[Ca]_i$ within prostatic cancer cells. Thapsigargin (TG) is a sesquiterpene lactone derived from the Thapsia Garganica plant which is a potent inhibitor (i.e. $IC_{50}$~10 nM) of the Sarcoplasmic/Endoplasmic Reticulum $Ca^{2+}$ A Tpase (SERCA) pump within the ER of cells. Such TG induced SERCA pump inhibition results in the depletion of the pools of $Ca^{2+}$ within the ER which generates a signal for the plasma membrane to open a capacitance channel to allow the influx of extracellular $Ca^{2+}$ into the cell. This results in an initial elevation of the $[Ca^{2+}]$ intracellular $Ca^{2+}$ $[Ca]_i$ from 20–40 nM to 200–400 nM. During this initial $Ca^{2+}$ rise, there are changes in gene expression with decreases in proliferation associated genes (e.g. cyclin D) and increases in apoptosis associated genes (e.g. GADD145 and IP3 type 3 receptor). After 6–8 hours, the $[Ca]_i$ returns to baseline value of 20–40 nM due to the activation of calmodulin dependent plasma membrane $Ca^{2+}$ pumps. After a variable interval of at least 12 hours, IP3 type 3 receptors within the plasma membrane form $Ca^{2+}$ channels allowing influx of extracellular $Ca^{2+}$ to produce a second (i.e. delayed) rise in $[Ca]_i$. This delayed rise involves externalization of annexin V protein. Once externalized, the annexin V protein oligomerizes to form a channel which allows further influx to elevate the $[Ca]_i$ to 2–15:M. This :M elevation activates calmodulin/calcineurin complexes allowing the dephosphorylation of the pro-apoptotic protein, BAD. Once dephosphyrylated, BAD translocates to the mitochondria allowing release of Cytochrome C, AIF, and SMAC. This activates the caspase and DNase cascade inducing the formation of apoptotic bodies.

While TG can induce the apoptotic death of androgen independent prostatic cancer cells, such induction is not targeted only to malignant cells. To target TG's apoptotic induction to prostatic cancer cells, primary amine containing TG analogs have been synthesized which have been covanlently coupled via a peptide bond to a specific amino acid sequence to produce a toxically inactive prodrug. The peptide sequence used was chosen so that it is hydrolyzed by the serine protease activity of prostate-specific antigen (PSA) thus liberating the toxic TG analog only in metastatic sites of prostatic cancer. This PSA targeted TG prodrug is presently being tested in pre-clinical models.

### S19 APPLICATIONS MICROARRAY TECHNOLOGY

CS Cooper, Institute of Cancer Research, Cotswold Road, Sutton, Surrey SM2 5NG, UK

**Abstract not received.**

### S20 HOW CAN PROSTATE-SPECIFIC GENE EXPRESSION PATTERNS BE EXPLOITED IN THE DEVELOPMENT OF TUMOUR GENE THERAPY?

NJ Maitland, YCR Cancer Research Unit, Dept of Biology, Univ of York, YO10 5YW, UK

Prostate cancer is a heterogeneous disease for which long-term survival is poor. Conventional therapies produce disappointing and heterogeneous responses and in its androgen independent bone metastatic form the tumour is virtually incurable. There is a pressing requirement for new therapies, but unless these therapies can be targeted to the tumour cells, they will offer no advantages. The prostate offers a variety of tissue and tumour specific targeting options, based on specific gene expression patterns, which can be harnessed to target gene-based therapies (Maitland 2000 Curr Opin Mol Ther 2:3).

Firstly, there are distinct changes at the cell surface of prostate cancer cells, such as adhesion molecules, growth factor receptors and a large number of unknown but recently catalogued changes (Liu, 2000 Cancer Res 60: 3429–3434). Many show differential expression between normal and malignant epithelium, but it is important to note that these changes are only seen in a proportion of individual tumours, and commonly in a proportion of cells within a single tumour mass. However attachment targeting will provide a selection advantage for therapeutic gene carriers, of viral or non viral origin, using either directly modified viral capsid or envelope proteins, or adaptor molecules, such as bispecific antibodies.

Perhaps more promising is transcriptional targeting. Expressed sequence tag (EST) analysis indicated that the basis of high level prostate restricted gene expression was at the transcriptional level. Both differential display, and now microarray analysis have identified numerous candidate genes whose promoters could be employed to direct gene therapy (Nelson et al, 2000 Nucleic Acids Res 28: 212–213). Some are obvious, such as prostate-specific antigen and membrane antigen, but others such as DD3 and prostate stem cell antigen also offer elements of targeting specificity, which could be employed either singly or in combination. We have demonstrated in a series of *in vitro* and *ex vivo* models that these promoters offer sufficient specificity and activity.

Finally, selection of therapeutic, genes can provide a third level of specificity. While prodrug activation by GDEPT strategies and specific stimulation of the immune response are common strategies now in clinical trial, there are also a number of genes, frequently inactivated in prostate cancers, whose reactivation could provide a necessary alternative. For example, the PTEN gene is frequently deleted and inactivated in prostate cancers. Restoration of PTEN activity is rapidly toxic for tumour cells, whereas normal epithelium is resistant.

Thus, a knowledge of the basic molecular properties of a heterogeneous and therapy-resistant tumour cell should provide the necessary specificity to provide effective and targeted gene therapy for prostate tumours.

### S21 EXCITABILITY OF PROSTATE CANCER CELLS – A NEW CONCEPT IN THE PATHOPHYSIOLOGY OF METASTATIC DISEASE

MBA Djamgoz, Department of Biology, Neuroscience Solutions to Cancer Research Group, Imperial College of Science, Technology & Medicine, London SW7 2AZ, UK

We have used a novel approach combining electrophysiological recording with cell and molecular biology to identify possible mechanisms distinguishing ‘slow’ from ‘fast’ progressing tumours of the prostate. Membrane potentials (which typically are some $10^7$ V/m), and associated ion channel proteins, are well known to exert a profound influence on various types of cellular behaviour, involved in the metastatic cascade, and abnormal ion channel expression (‘channelopathy’) has been demonstrated in numerous diseases.

**Electrophysiology** Single-cell patch clamp recordings from rat and human prostate cancer (PC) cell lines of strong vs weak metastatic potential (MAT-LyLu/AT-2 and PC-3/LnCaP cells) revealed voltage-gated $Na^+$ channels (VGSCs) expressed specifically in the strongly metastatic cells. These VGSCs belonged to the tetrodotoxin (TTX)-sensitive class. In addition, the strongly metastatic cells expressed significantly smaller $K^+$ currents. Taken together, these electrophysiological characteristics would render the strongly metastatic cells electrically excitable.

**Molecular biology** Multiple VGSC α-subunit genes were found to be expressed at very low levels in both the strongly and the weakly metastatic rat and human PC cell lines. However, one particular VGSCα gene, SCN9A, occurred at a level approximately 1000 times greater in strongly *vs* corresponding weakly metastatic cells. This VGSCα, which would indeed give rise to TTX-sensitive currents, could be responsible for the functionally detectable $Na^+$ currents in these cells.

**Functional studies in vitro** VGSC activity could directly enhance metastatic ability by potentiating important cellular behaviours integral to the metastatic cascade, including process extension, lateral motility, secretory membrane activity, directional movement in an electric field (galavanotaxis) and transverse invasion.

**VGSC expression in vivo** Immunohistochemical staining showed that expression of VGSC protein also occurred in sections human PC in vivo. The expression levels were very weak for benign prostatic hyperplasia and low-grade prostatic intraepithelial neoplasia (PIN). For high-grade PIN and in situ malignancy, however, VGSC expression appeared significantly higher.

These results are consistent with the hypothesis: 1. that a basal level of VGSCα expression, which occurs in weakly metastatic cells, is greatly upregulated in the strongly metastatic phenotype; 2. that it is predominantly the SCN9A gene which is responsible for this upregulation, by a presently unknown mechanism(s); and 3. that the associated VGSC activity is an accelerating factor in PC. Accordingly, the SCN9A gene and/or its products can potentially represent a new marker for distinguishing ‘slow’ from ‘fast’ progressing (low- vs high-metastatic) PC.

### S22 SCREENING FOR CANCER: IMPACT ON MORTALITY AND IMPLICATIONS FOR HEALTHCARE DELIVERY

P Boyle, European Institute of Oncology, Milan, Italy and ICRF, UK

Screening has the potential to make a greater contribution to reducing national cancer mortality rates. Progress is being made both in the development of better screening tests and in introducing screening programmes into the general population in an effective manner. Screening for cervix cancer with the Papanicolau smear has been effective at reducing both the incidence of invasive cancer and, consequently, mortality from cervix cancer. A plateau of what can be accomplished may now have been reached and the role of simultaneous HPV testing is becoming a focus of research activity. Mammographic screening for breast cancer is effective in randomised trials in reducing mortality from breast cancer, at least among women aged 50–64. Recent declines in breast cancer mortality in the UK appear to be at least partially due to the implementation of a National Breast Cancer Screening Programme in the late 1980s. Faecal Occult Blood Testing (FOBT) has now been shown to reduce mortality from colorectal cancer while the results of randomised trials of screening with sigmoidoscopy are eagerly awaited.

There are several outstanding areas of great controversy. There are proponents of using prostate-specific antigen (PSA) screening to reduce the mortality from prostate cancer: results of two large randomised trials will become available in (approximately) 4 years.

Ovarian cancer is also an area of screening activity and a large randomised trial has been established in the UK to investigate this issue. There are on-going debates about screening for oral cancer and stomach cancer which need to be addressed. Issues surrounding genetic screening are only beginning to be touched.

The importance of screening lies in having effective strategies implemented. Screening research and organisation of screening programmes should be a shared activity between the research community and NHS policy makers. At a time when we are making progress in Cancer Control, closer collaboration on screening has the potential to improve health and further reduce premature mortality.

### S23 WILL HISTOPATHOLOGY BE REPLACED BY MACHINES?

B A Gusterson, Dept. of Pathology, Glasgow University, Glasgow G11 6NT, UK

Histopathology is based in morphology and this will continue for the foreseeable future. It is significant that at the recent NIH/NCI review of the management of early breast cancer that the consensus was that none of the findings in relation to the biochemical or molecular analysis of breast cancer had demonstrable application in the clinical decision making process. Once the age of the patient, size of the tumour, morphological grade, lymph node status and hormone receptor status were taken into account, everything else had no clinical utility for prognostic or predictive testing. The major problem has been that there has been a total lack of quality control in the application and analysis of new ‘markers’. Major advances have been made in understanding mechanisms of carcinogenesis in many tumour types, but in most instances this has lead to an increased awareness of the complexity of the process. It is clear that primary genetic changes can predetermine morphology, but secondary changes and epigenetic changes lead to a complex phenotype with every tumour accumulating shared, but an overall unique set of abnormalities. It is the ability of the pathologist to optically ‘compute’ these changes based on decades of experience and proven good clinical correlation that has resulted in the present NIH/NCI statement. It is now for the scientific community to take on the challenge and to demonstrate that the new technologies afforded by the genomic and proteomic revolution have more to offer that has been suggested by the recent collapse in technology share prices. In terms of pathology there is no doubt that microarray analysis and protein profiling is in its infancy and has an exciting future. Whether these techniques will replace pathology as a diagnostic and predictive tool will depend largely on an active interface between pathologists and the scientific community with an emphasis on quality control and statistics input to study design. There is no doubt that cautious scepticism is warranted with a reasonable prediction that diagnosis will be the remit of the morphologist, linked with some form of biochemical/molecular analysis to predict tumour behaviour and treatment efficacy.

### S24 CLINICAL VALUE OF PCR DETECTION OF CIRCULATING TUMOUR CELLS

Selby P, Imperial Cancer Research Fund Clinical Centre in Leeds, Cancer Research Building, St James’s University Hospital, Beckett Street, Leeds LS9 7TF, UK

Despite the successes of nucleic acid amplification techniques in the evaluation of lymphomas and leukaemias, for solid tumours progress has been much slower because of the absence of specific markers and more difficult access to tumour sites. The suggestion that the detection of tissue specific gene expression using RT-PCR might be an alternative approach for solid tumours and would be sufficiently sensitive to be applicable to peripheral blood was first mooted 10 years ago. Experiments to prove the principle and to pilot a number of targets particularly tissue specific enzymes but also cytokeratins and prostate specific antigen were carried out. The principle is sound and it is possible to demonstrate the presence of circulating tumour cells using RT-PCR to detect gene expression for solid tumours using target genes which are not expressed in blood cells.

The technology has proved difficult with problems including easy contamination, instability of RNA. Consistent quality has been difficult to assure especially between different laboratories.

Nevertheless, it has been possible to demonstrate clinically significant findings. Peripheral blood cells detected in this way are associated with higher stage and poor prognosis disease. This is illustrated by recent studies in neuroblastoma in which positive RT-PCR to detect mRNA for tyrosine hydroxylase has been shown to be an independent prognostic factor after multivariate analysis predicting the outcome for children with neuroblastomas.

New technologies to replaces RT-PCR have been explored including nucleic acid sequence based amplification (NASBA) but have yet to be shown to be more robust or more sensitive than RT-PCR for this purpose. Studies with NASBA and direct comparisons to RT-PCR both in model systems and clinical samples illustrate this point.

This approach can yield data of clinical significance and perhaps potential clinical utility. However, the technological problems are such that it is unlikely yet to become a routine staging investigation for solid tumours.

### S25 THE DEVELOPMENT & APPLICATION OF IN VIVO PHARMACODYNAMICS & PHARMACOKINETIC ENDPOINTS USING PET IN EARLY ANTI-CANCER CLINICAL TRIALS, PM Price, Academic Dept of Radiation Oncology, Christies Hospital NHS Trust, Manchester M20 4BX

Positron emission tomography (PET) uses radio-nuclides to label molecules which then can be imaged in man, providing quantitative kinetic information. The inherent sensitivity and specificity of PET is unrivalled. PET can image molecular interactories and pathways, providing quantitative kinetic information down to the sub picomolar levels. This technology has the potential to answer a large number of important clinical questions in oncology translational research. However, the challenges in the methodology are substantial. The CRC/MRC Programme has been developing molecular imaging using PET to assess in vivo pharmacokinetics and pharmacodynamics.

**Pharmacodynamics** The CRC Phase 1 study of Cambretastation has been paralleled with PET measures of physiological vascular parameters. The timing, magnitude and mechanism of action of the compound, has been demonstrated in man. In vivo tumour proliferation can now be measured with 2 (c) thymidine. Methodology is being developed to quantitate in vivo signal transduction and other pharmacodynamic end points.

**Pharmacokinetics** Novel generic strategies for carbon 11 and fluorine 18 labelling of anti-cancer molecules have been developed. Labelled compounds have been used in early clinical trials, to provide in vivo tumour and normal tissue pharmacokinetic data.

As PET methodologies develop further, so this technology can be more rapidly applied to drug development studies and extended to other therapy assessments, such as radiation response.

### S26 NON-INVASIVE, QUANTITATIVE AND REPETITIVE IMAGING OF REPORTER GENES IN LIVING INDIVIDUALS

HR Herschman, J Barrio, N Satyamurthy, M Phelps, Q Liang, D MacLaren, S Gambhir, Crump Institute for Molecular Imaging, University of California, Los Angeles CA 90095, USA

We have developed methods to quantitatively and non-invasively measure reporter gene expression in living subjects. We use positron emission tomography (PET) to measure the sequestration of positron labelled chemical probes that are only trapped in tissues if ‘PET reporter genes’ are ectopically expressed.

One of the genes we use as a PET reporter gene encodes the Herpes Simplex virus thymidine kinase gene. HSV-TK, like cellular TKs, can phosphorylate thymidine. HSV1-TK can also phosphorylate acycloguanosine thymidine analogues (acyclovir, ganciclovir, penciclovir). We use flourine 18 (positron-emitting) labeled versions of GCV and PCV as ‘PET reporter probes’ to detect ectopic expression of HSV1-TK. We have optimised this ‘PET reporter gene/PET reporter probe’ imaging system by both synthesizing alternative substrates and by using a mutated HSV1-tk gene.

We have developed a second PET reporter gene system that employs the dopamine D2 receptor as the PET reporter gene and a positron-labeled version of spiperone (a dopamine antagonist) as the PET reporter probe. The D2R receptor is expressed primarily in the striatum, thus lending itself for use as a reporter gene in other areas of the body.

We have validated the use of PET reporter gene/PET reporter probe imaging technology in both somatic gene delivery systems and in transgenic mice, using a dedicated small animal PET scanner, the microPET. This technology is able to determine the location, magnitude and duration of reporter gene expression in a non-invasive, quantitative and repetitive fashion. We anticipate the major clinical use of the PET reporter gene technology will be in “tracking” the location magnitude and duration of gene expression following induction of DNA delivery vectors carrying combinations of therapeutic genes of PET reporter genes.

### S27 THE ROLE OF MAGNETIC RESONANCE IMAGING IN BREAST CANCER RESEARCH FJ Gilbert, Dept of Radiology, University of Aberdeen, Lilian Sutton Building, Foresterhill, Aberdeen AB25 2ZD, UK

The advent of high-resolution magnetic resonance imaging (MRI) combined with rapid data acquisition following intravenous contrast administration has allowed a more functional approach to breast cancer diagnosis and follow-up. MRI as a more accurate pre-operative staging tool in terms of tumour size and multicentricity is being assessed to determine whether reoperation rates and recurrent disease can be reduced. Both gadolinium DTPA and USPIO iron oxide particles have been used to determine lymph node status preoperatively. Neoadjuvent chemotherapy regimes can be evaluated by monitoring changes in k surface area permeability product and $v_L$ interstitial fluid and 3D assessment of tumour size. The same methods can be used to monitor other novel preoperative approaches such as Hyperbaric oxygen treatment, antiangiogenesis therapies and also to gain further understanding in the relationship between vascularity and tumour metabolism by combining MRI data with PET over serial examinations. These methodologies provide exciting opportunities for evaluation of novel chemotherapeutics. New macromolecular contrast agents for blood pool imaging, molecular targeting and fluorescent probes are being developed which will open new avenues of research.

Research is ongoing as to the value of MRI in determining residual disease, recurrent disease and in occult disease. Large multicentre screening studies are continuing in women with a strong family history of breast cancer.

### S28 RHO-LIKE GTP-ASES IN CELL ADHESION, CELL MIGRATION, AND TUMOUR FORMATION JG Collard, The Netherlands Cancer Institute, Division of Cell Biology, Plesmanlaan 121, 1066 CX Amsterdam, The Netherlands, Supported by the Dutch Cancer Society

Rho family proteins have been implicated in the control of a wide range of biological processes, which include signaling pathways that regulate the actin cytoskeleton in response to receptor stimulation. We have identified the Tiam1 gene, which encodes an activator (GEF) of the Rho-like GTPase Rac and induces invasion in T-lymphoma cells.

In epithelial carcinoma cells, invasion and metastasis is associated with loss or reduced E-cadherin-mediated cell–cell adhesion. Tiam1 co-localizes with E-cadherin in epithelial cell–cell contacts. Ectopic expression of Tiam1 inhibits HGF-induced cell scattering and cell migration by increasing E-cadherin-mediated cell–cell adhesions. In addition, Tiam1-Rac signaling inhibits invasion and migration of fibroblastoid Ras-transformed MDCK cells by restoring E-cadherin-mediated adhesions and an epithelial phenotype. Tiam1/Rac-induced cellular responses with respect to cell-cell adhesion and cell migration are also dependent on integrin-mediated cell substrate interactions. Our data indicate that invasion and migration of epithelial cells is determined by a balance between invasion-inhibitory, E-cadherin-mediated, cell-cell interactions and invasion-promoting, integrin-mediated, cell-substrate interactions. Both processes are regulated by Rac and Rho proteins. Furthermore, Rac and Rho play antagonistic roles in epithelial-mesenchymal transition. This transition is required for the acquisition of an invasive and metastatic phenotype of epithelial tumors. Activation of Rac promotes cell spreading and firm cadherin-based cell-cell adhesions whereas activation of Rho leads to cell contraction and a mesenchymal migratory phenotype. We found that Rac is able to downregulate Rho activity directly at the level of the GTPase and that the reciprocal balance between Rac and Rho activity is a major determinant of epithelial-mesenchymal transition. In spite of a large number of in vitro data, little is known how Rac and Rho affect invasion and metastasis of epithelial tumors in vivo. Currently we are studying how deregulated Rac signaling influences invasion and migration of epithelial tumors by making use of Tiam1 knock out mice.

Supported by the Dutch Cancer Society.

### S29 THE ROLE OF ADHESION-LINKED TYROSINE KINASES IN ONCOGENESIS

M C Frame, The Beatson Institute for Cancer Research, CRC Beatson Laboratories, Garscube Estate, Switchback Road, Bearsden, Glasgow G61 1BD, UK

The development of malignancy is associated with de-regulation of cell-matrix and cell-cell adhesion, often mediated by altered expression or function of adhesion components, including the integrin and cadherin families of cell surface receptors. Alterations to intracellular signalling molecules that control the adhesion assembly/disassembly cycle, or adhesive strength, can also contribute to cancerous behaviour. Included in this group are the Src and FAK families of intracellular tyrosine kinases, members of which are located at integrin-linked focal adhesions in fibroblasts, and, in the case of Src, also at cell-cell contacts in epithelial cells. Our work on fibroblast transformation by Src oncoproteins has indicated a) how its intracellular targeting to cell-matrix adhesions is controlled, and b) how phosphorylation of its substrates, including FAK, initiates signalling into the cell interior resulting in promotion of integrin adhesion turnover that is required for cell motility and invasion.

In epithelial cells, Src inhibition results in the cadherin-dependent stabilization of cell-cell contacts. As a consequence, cells are unable to dissociate from an epithelial sheet and migrate or invade. Thus, inhibiting the Src family kinases, or their downstream effectors, may have therapeutic benefits by slowing down tumour spread. Since the expression and/or activity of Src is elevated in colon cancer, and since current treatments for advanced colon cancer are only poorly effective, we are studying what role Src plays and identifying its epithelial binding partners and substrates in colon cancer cells. In addition to identifying Src's role, we are using genetic manipulation in mice to examine the role of FAK in cancer development and progression *in vivo*. The aims are to optimise interventionist strategies and to establish whether inhibiting Src- or FAK-dependent events will suppress cancer cell invasion or metastasis.

### S30 GROWTH FACTOR SIGNALING IN CARCINOMA PROGRESSION

JP Thiery, F Radvanyi, J Jouanneau, CNRS UMR 144 Institut Curie, 26 rue d'Ulm 75248 Paris, Cedex 05, France

The plasticity of epithelial cells is of critical importance during embryogenesis. Epithelial cell plasticity may also be involved in carcinoma progression. We have studied a malignant bladder epithelial cell line (NBT-II) that can undergo reversible conversion to motile fibroblastic-like cells (epithelium-mesenchyme transition, EMT) upon exposure to several growth factors. Scatter activity involves early and transient activation of c-Src. Conversion also requires activation of the Ras-MAP kinase pathway. Slug, a transcription factor related to snail in *Drosophila*, is also induced early, before the dissociation of epithelial cells. Snail is a zinc finger transcriptional repressor produced during early gastrulation in invaginating mesodermal cells. Cells producing slug lose their demosomes and are converted into stationary fibroblast-like cells. NBT-II epithelial cells can also undergo EMT upon exposure to native collagens and laminin 5. Activation of the $\alpha 2\beta$ l integrin by type 1 collagen induced specific tyrosine phosphorylation of FAK and paxillin. Transient expression of a paxillin gene with mutations affecting two critical tyrosines strongly inhibited NBT-II cell motility on a collagen-coated substrate. We will present a model summarizing our current view of the mechanisms governing EMT in NBT-II cells. We have investigated whether epithelial cell plasticity is involved in tumor progression by analyzing the behavior of NBT-II cells expressing growth/scatter factors constitutively or following induction. Thiery, Chopin, 1999 Cancer Metast Rev 18: 31–42. FGF-1-expressing cells have a fibroblastic morphology in vitro and were much more tumorigenic in nude mice than mocked transfected NBT-II cells. The role of FGF-2 was also investigated in NBT-II cells rendered autocrine for this growth factor. Autocrine behavior was required for NBT-II cells to become invasive, angiogenic and tumorigenic. NBT-II cells expressing a high-molecular mass isoform of FGF-2 became highly metastatic in the lung via a FGF receptor-independent pathway. Recent studies of human bladder carcinoma have demonstrated the multifunctional properties of some growth factors, which act positively via enhanced autocrine loops or negatively, as suppressors of tumor progression. This work clearly shows that similar mechanisms control epithelial morphogenesis in development and tumor progression (Cappellen et al, 1999 Nat Genet 23: 18–20)

### S31 PHOSPHOHEXOSE ISOMERASE/AUTOCRINE MOTILITY FACTOR/NEUROLEUKIN/MATURATION FACTOR IS A MULTIFUNCTION PROTEIN

A Raz, Karmanos Cancer Institute, 110 East Warren Avenue, Detroit, MI 48201, USA

Phosphohexose isomerase (PHI) is a member of the ectoenzyme/exoenzyme family and plays a key role in both glycolysis and glucogenesis pathways. Upon secretion PHI acts as a cytokine with tumor autocrine motility factor (AMF), neuroleukin (NLK) and maturation factor (MF) functions. Signalling is initiated by its binding to a cell surface 78 kDa glycoprotein (gp78). The receptor contains a seven transmembrane domain, a RING-H2 motif and a leucine zipper motif. Analysis of the amino acid sequence of gp78 with protein databases revealed no significant homology with all known seven transmembrane proteins, but a significant structural similarity to a hypothetical protein of Caenorhabditis elegans, F26E4.11. We analysed cytoskeletal rearrangements following the AMF stimulus, and found an extensive and early (5–15 min) formation of stress fibers and accumulation of cortical actin. It was followed by rearrangements of PY+ adhesion plaques to the poles of the stress fibres and translocation of PKC both to stress fibers and to cortical actin associated with up-regulation of RhoA and Rac1 expression with no change in the expression of Cdc42. Treatment of the cells with C3 exoenzyme, prior to AMF stimulation inhibited the formation stress fiber-like structures and the cells did not respond by stimulation of expression of RhoA. In addition, the two isoforms of JNK; JNK1 and JNK2, were concomitantly activated by AMF, supporting the notion that both are involved in the signalling pathway of RhoA. Suggesting that autocrine motile signalling shares a similar pathway to the previously established paracrine signalling, involving cytoskeletal rearrangement and morphological alterations mediated by the small RhoA-like GTPases.

GP78 expression has already been suggested as a prognostic indicator for use in the bladder and colorectal cancer, and the expression of E-cadherin is down-regulated in response to an AMF-like molecule produced by oral squamous cell carcinoma cells as well. Additional studies to investigate the generality of the findings of this study with regard to other tumor cell systems and the use of gp78 as a possible marker for loss of cell contact regulation in addition to being a growth/motility modulator in epithelial cells that is associated with down-regulation of cell-cell adhesion molecules should not only aid in predicting tumor prognosis but also lend insight into the factors associated with the acquisition of motility and the malignant phenotype by tumor cells.

### S32 GENE EXPRESSION PROFILING FOR CLASSIFICATION AND PROGNOSIS OF CHILDHOOD SOLID TUMOURS

T Triche, Children's Hospital, Los Angeles, USA

**Abstract not received.**

### S33 BIOLOGICAL AND CLINICAL IMPACT OF CHROMOSOMAL TRANSLOCATIONS IN CHILDHOOD LEUKAEMIA

M Greaves, LRF Centre, Institute of Cancer Research, London SW3 6JB, UK

Chromosomal translocations in leukaemia produce illegitimate recombinants which come in two flavours: either expression of one gene is dysregulated by its new juxtaposition to constitutively active promoters (e.g. for *IG* or *TCR*) or a novel, chimaeric gene is formed. The latter variety predominates in the acute leukaemias and the resultant product usually has activated kinase activity or altered transcriptional regulation activity. The predominant mechanism of fusion gene formation appears to be error-prone non-homologous end joining but *IG/TCR* recombinase signal sequences or topo II sites may also be involved in special cases. Breaks occur clustered in intronic regions of fusion genes and each patient's leukaemic clone has a unique or clonotypic breakpoint. This then provides a specific, sensitive and stable molecular marker for tracking the clone. Applying these PCR-based markers to monozygotic twins with concordant leukaemia and retrospective scrutiny of neonatal blood spots has revealed that gene fusions that are common in paediatric leukaemia arise predominantly in utero. For infants, *MLL* fusion gene formation may lead to leukaemia without further exposures after a very short latency. The same applies to secondary leukaemias induced by chemotherapy. For childhood leukaemia (with *TEL-AML1* and *AML1-ETO* fusions), additional post-natal events are required; latency is correspondingly variable and protracted, as also indicated by transgenic models. Recent case/control molecular epidemiological studies have shed some light on the interactions between inherited genetic variability at a number of loci and environmental exposures that may cause these genetic alterations.

Clinically, the presence of particular chromosomal translocations may be predictive of outcome and be of value for differential diagnosis, prognostication and monitoring of residual disease. Recent insights into the foetal origins of leukaemia fusion genes and the natural history of the disease may also have implications for the clinical pattern of remission and relapse. In particular, translocation-bearing foetal clones of putative pre-leukaemic cells may survive chemotherapy and contribute to late or 'off-treatment' relapse.

### S34 BIOLOGICAL AND CLINICAL IMPACT OF CHROMOSOMAL TRANSLOCATIONS IN CHILDHOOD SARCOMAS

M Ladanyi, Department of Pathology, Memorial Sloan-Kettering Cancer Center, New York, NY, USA

Several paediatric sarcomas are characterized by specific recurrent chromosomal translocations. Since 1992, the breakpoints of almost all of these translocations have been cloned, revealing that they produce highly specific gene fusions, usually encoding aberrant chimaeric transcription factors. While these tumours may individually be relatively uncommon, they are of special scientific interest because their specific translocations pinpoint the oncogenic lesion central to their pathogenesis, thereby making them considerably more tractable for further analysis than other solid cancers, such as carcinomas. On the diagnostic side, the specificity of these translocations, along with the relative ease with which can be tested for, and the frequent histopathological difficulties associated with these sarcomas, have thrust this group of tumours to the forefront of solid tumour molecular diagnostics, second only to *NMYC* amplification testing in neuroblastoma in terms of clinical acceptance. Furthermore, fusion gene detection has confirmed and refined the nosology of several sarcoma groups. Besides the cloning of chromosomal translocations and their diagnostic applications, several groups have examined the clinical and biological impact of structural variability of these gene fusions, and the remarkable prognostic impact of secondary genetic alterations in these sarcomas with gene fusions. Such studies have led to plans by European and American cooperative groups to evaluate these molecular prognostic markers in the next generation of Ewing's sarcoma clinical trials. Finally, translocation-associated paediatric sarcomas are also emerging as ideal candidates for evaluating new approaches such as expression profiling by cDNA microarrays and fusion protein-directed cancer immunotherapy. Thus, work from multiple groups over the past decade on these tumours has provided insights into their basic pathobiology and pathologic classification, and has identified molecular targets for diagnostic, prognostic, and therapeutic applications.

### S35 TARGETING NUCLEAR PROTEIN COMPLEXES IN CANCER CELLS FOR SELECTIVE CANCER THERAPEUTICS

H Hurley, College of Pharmacy and Arizona Cancer Center, University of Arizona, Tucson, Arizona, USA

Small molecules that cause specific transcriptional inactivation, interfere with molecular switching mechanisms, are molecular architectural enforces, may be used in protein hijacking or lethal molecular engineering provide attractive opportunities for modern cancer therapeutics. What may be surprising about these terms is they have all been applied to agents that target DNA, usually in combination with DNA binding proteins. In this presentation I will trace the development of ideas that make DNA a powerful ally in strategies that lead to selective therapeutics. While the emphasis will be on strategies that take advantage of secondary DNA structures formed by unique sequences of DNA in critical regions of cancer cells, I will also discuss examples of agents that gave rise to these more sophisticated concepts. Much of what will be described will be taken from my laboratory and will include compound such as ecteinascidin 743, which has a unique mechanism of action, involving nuclear excision repair-dependent uncoupled incisions, and compounds that trap out defined conformational forms of DNA. Supported by grants from the National Cancer Institute.
